# Age and Physical Activity Modulate the Spatial Mapping of Time-Related Words

**DOI:** 10.5334/joc.514

**Published:** 2026-09-04

**Authors:** Anastasia Malyshevskaya, Martin H. Fischer, Yury Shtyrov, Andriy Myachykov

**Affiliations:** 1 Cognitive Sciences, University of Potsdam, Potsdam-Golm, Germany; 2 Cognitive Health and Intelligence Center, Institute for Cognitive Neuroscience, HSE University, Moscow, Russian Federation; 3 Center of Functionally Integrative Neuroscience, Department of Clinical Medicine, Aarhus University, Aarhus, Denmark; 4 Centre for Cognitive and Brain Sciences, University of Macau, Taipa, China

**Keywords:** spatial cognition, attention, mental time line (MTL), ageing, physical activity

## Abstract

The processing of temporal concepts is known to be intertwined with spatial cognition. For instance, reaction times (RTs) are shorter when participants classify past- and future-related words with left- and right-lateral responses, respectively, in line with the so-called *mental time line* hypothesis. While some research has addressed individual differences in processing temporal concepts, it still remains unclear how such space–time congruency effects are influenced by the most crucial time-related factor in our lives – age. To address this question, we asked 94 participants aged 19 to 80 years old to classify past, future, and neutral words as related to time by using left and right response keys. In addition, we collected information regarding participants’ self-reported level of physical activity, as it was shown to be a predictor of healthy ageing. We found that younger participants processed future-related words faster than past-related words, whereas older participants processed past-related words faster than future-related words. This ageing effect was modulated by physical exercise: The higher the self-reported level of physical activity in older participants, the smaller the past-related RT bias. Finally, our data revealed an age-related decline in space–time congruency effects among participants with lower self-reported physical activity. These findings indicate that the conceptual representation of time undergoes lifelong changes influenced by multiple factors, including age and physical activity. We suggest an integrative construct of *dynamic conceptual time* whereby the individual’s bodily and life-course experiences lead to gradual changes in the processing of temporal concepts.

*“The ordinary experiences of aging alter and clarify your view of past, present and future.”*

Edith Pearlman (Pearlman, 2011)

## 1. Introduction

The ability to orient oneself in time is an essential feature of cognition, interfacing with perception, attention, memory, and decision making (Block & Gruber, 2014; Hinault et al., 2023; Matthews & Meck, 2016; Nobre & van Ede, 2018). A central dimension of temporal cognition is *mental time travel* – the ability to mentally navigate past, present, and future events – which plays a crucial role in goal pursuit, self-identification, and self-continuity (Becker et al., 2018; Buzi et al., 2024; Conway et al., 2019; D’Argembeau et al., 2008). Unlike other senses, time has no known dedicated perceptual receptors, and the experience of time is inherently subjective (Heidegger, 1962). Here, we investigate how attention shapes the conceptualisation of time, with a particular focus on how individual experiences – ageing and physical activity – modulate this process.

Mental time travel (Suddendorf & Corballis, 1997; Tulving, 1985) enables us to mentally project oneself into the past or future, drawing on episodic and semantic memory as well as broader cognitive functions (Irish & Piguet, 2013; La Corte & Piolino, 2016; Schacter & Addis, 2007). The neural network underlying this capacity is believed to be widely distributed, encompassing the hippocampus, prefrontal cortex, and parietal regions (Hinault et al., 2023; Viard et al., 2011), and develops sufficiently by ages 3–4 to support basic temporal reasoning (Buzi et al., 2024). Because temporal concepts are highly abstract and lack dedicated receptor-type mechanisms, their representation is thought to rely on grounding in other modalities – particularly spatial ones – as proposed by embodied approaches to cognition (Barsalou, 2008; Myachykov et al., 2014). This is evidenced by, e.g., the use of spatial language for time (future is “ahead”, past is “behind”), spatially directed gestures employed to describe time, and impaired past-reasoning in individuals with hemispatial neglect (Bonato et al., 2016; Boroditsky, 2000; Núñez & Cooperrider, 2013).

A key empirical signature of this space–time grounding is the *Space-Time Congruency Effect*: responses to time-related words are faster when past maps to the left and future maps to the right in an experimental task (von Sobbe et al., 2019). This is broadly consistent with a horizontal *Mental Time Line (MTL)* — a left-to-right representation of time — such that processing temporal concepts triggers spatial orienting analogous to attentional cueing (Posner, 1980; Shaki & Fischer, 2023). It is important to note, however, that space–time congruency effects are not unambiguously interpretable as direct evidence for an activated spatial representation of time. Recent work has shown that these effects can be fully accounted for within stimulus–response compatibility frameworks, without invoking obligatory attentional shifts along a mental time line (Scozia et al., 2023a, 2023b, 2024). Spatial coding of time appears preferentially at slower response latencies and depends on how spatial and temporal codes are jointly recruited by task demands – possibly suggesting that space may function as a late heuristic rather than an intrinsic dimension of time representation. In light of this, we will interpret our congruency findings cautiously, as reflecting space–time compatibility at the level of response selection.

MTL representation varies systematically with language and experience. Reading direction shapes the left-to-right *horizontal MTL* in speakers of European languages (Bender & Beller, 2014; Bonato et al., 2012), whereas vertical metaphors and writing direction give rise to a *vertical MTL* in Mandarin speakers (Fuhrman et al., 2011; Gu et al., 2017; Ouellet et al., 2010a), and sensorimotor experience of forward movement underlies a *sagittal MTL* linking past with backward and future with forward space (Myachykov et al., 2014; Núñez & Cooperrider, 2013). Beyond such influences, multiple *individual differences* in MTL activation are also evident: for instance, the perceived location of the future varies with optimism, anxiety, religious beliefs, and political attitudes (Li & Cao, 2020, 2022; Li, 2023).

Despite growing interest in individual differences in MTL activation, little attention has been paid to how MTL changes with age, which in itself epitomises what is probably the strongest influence of time on human lives. Ageing is known to shift individual temporal perspective from future to past: from around age 50, people report a shrinking future horizon and increasing focus on the experienced past (Kooij et al., 2018; Strough et al., 2016). This generally coincides with a neurocognitive decline affecting memory and executive function (Fjell & Walhovd, 2010; Lister & Barnes, 2009), which may impair mental time travel ability per se. Indeed, older adults have been shown to generate fewer episodic details for both past and future events and to show reduced future projection (Addis et al., 2008, 2010; Spreng & Levine, 2006).

Importantly, age-related cognitive decline extends beyond memory and executive function to temporal processing more directly, with older adults showing systematic changes in duration perception and interval timing (Maaß et al., 2022; Mioni et al., 2021, 2024). Furthermore, older adults show altered patterns of spatial–temporal associations compared to younger adults, with differences emerging in both the magnitude and direction of congruency effects (Bogon et al., 2024; Hallez & Balcı, 2024; Jagorska et al., 2026). At the lexical level, Bottini and Casasanto (2010) demonstrated that spatial context systematically influences the interpretation of temporal language, yet this line of work has remained largely separate from the ageing literature. The present study sits at the intersection of these strands, examining how age modulates the spatial grounding of temporal word processing.

Importantly, ageing has been shown to modulate the sagittal MTL: contrary to the typical pattern, older adults are more likely to place the past in front of them, consistent with a past-oriented temporal focus (Bylund et al., 2020; de la Fuente et al., 2014). Furthermore, Anelli and colleagues (2016) found that older adults were slower than younger ones when classifying events under a future self-location condition on the horizontal MTL, suggesting a generally reduced capacity for future self-projection. No prior MTL study, however, has directly assessed age-related changes in horizontal space–time congruency effects. The present study addresses this gap by examining how age and self-reported physical activity jointly affect processing speed and horizontal spatial mapping of temporal words. Drawing on the findings discussed above, we expect horizontal space–time congruency effects to be weaker in older participants, who may also be generally slower to process future- than past-related words.

Furthermore, one putative moderating factor we consider alongside the age differences is physical activity, which has been shown to support episodic memory, executive control, and future time perspective in older adults (Aghjayan et al., 2022; Hamm et al., 2024; Kooij et al., 2018). In the present study, we treat self-reported physical activity as a proxy for general health and well-being, predicting that higher activity will be associated with a more preserved pattern of temporal cognition in older participants. Age and physical activity were expected to exert opposing effects: ageing shrinks the future horizon and shifts temporal orientation toward the past, while physical activity supports future time perspective and general cognitive health.

We tested three hypotheses. First, space–time congruency effects would be weaker in older than younger participants, reflecting reduced mental time travel capacity (cf. Anelli et al., 2016; Coste et al., 2012). Second, younger participants would process future-related words faster than past-related words, with the pattern reversing in older participants. Third, higher physical activity would be associated with stronger congruency effects and smaller age-related differences in past versus future word processing, particularly in older adults.

## 2. Methods

### 2.1. Participants

The sample size estimation was based on previous studies, which also focused on the space-time association using a semantic time classification task. In the meta-analysis by von Sobbe and colleagues (2019), 41 participants were indicated as a sufficient number to detect a medium-size (*d* = 0.46) space-time congruency effect. Similarly, in the study by Beracci and Fabbri (2022), the number of 41 participants was calculated as the minimum sample size. However, our design included two continuous predictors: participants’ age and the self-reported level of physical activity (see in detail in Experimental design and materials). Therefore, in order to increase the chance of detecting the space-time congruency effect, our sample size was extended to 94 participants.

**Figure 1:**
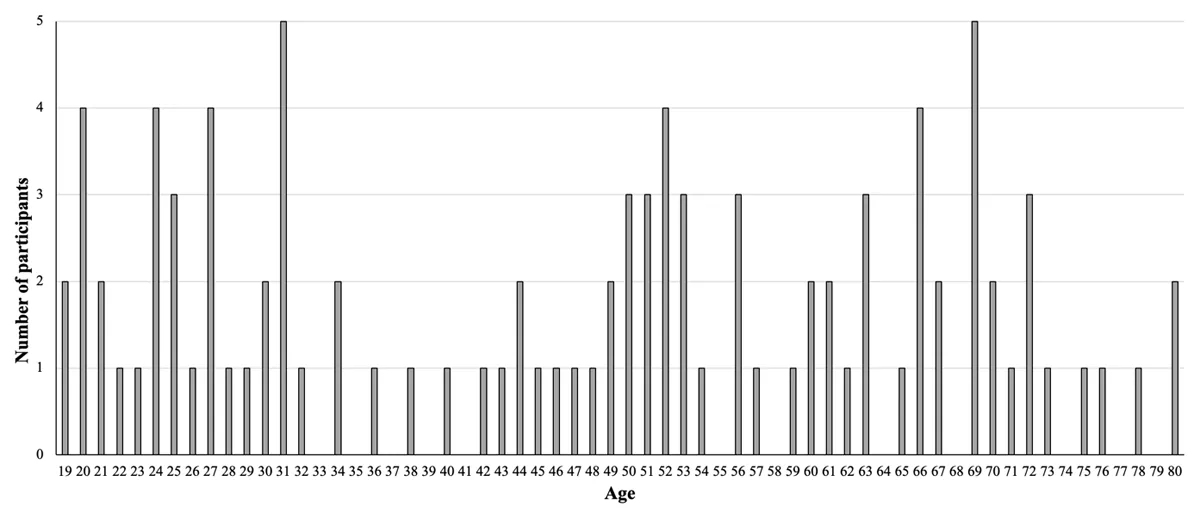
Age distribution.

We recruited participants of a wide age range (mean age 47.5 ± 18.3 years, ranging from 19 to 80 years old, 73 females) (see Figure 1). All participants were right-handed (self-assessed) native Russian speakers. Participants of all ages were recruited through various social media platforms. More senior participants (50 years old and above) were also recruited via the “Moscow Longevity” project (“Московское долголетие”; https://www.mos.ru/city/projects/dolgoletie/), which offers a variety of health, educational, and recreational programs to senior residents. All participants had no prior knowledge of the study design or hypotheses, were financially compensated for their time (250 rubles), and debriefed at the end of the session.

### 2.2. Experimental design and materials

After signing an informed consent form, participants completed a demographic questionnaire providing information regarding their age, gender, handedness, and their self-reported frequency of physical exercise (as a proxy for general health and well-being). Regarding the latter, participants had to describe the frequency of their exercise (“How often do you engage in physical activity?”; 1 – never, 2 – several times per year, 3 – several times per month, 4 – once per week, 5 – several times per week, 6 – every day). We acknowledge that this single-item measure is a coarse proxy and may be subject to individual differences in how “physical activity” is interpreted (e.g., gym-based exercise vs. everyday walking).

The main experimental stimuli consisted of 24 time-related words and 24 control neutral words of the participants’ native language (see Table 1). Neutral control words had no explicit time-related, spatial, or numerical meanings (e.g., *факультет – faculty, экономика – economics*). Time-related stimuli included 12 past- (e.g., *вчера – yesterday, издавна – since long ago*) and 12 future-related words (e.g., *завтра – tomorrow, предстоящий – forthcoming*). The latter stimulus set has previously been shown to produce a reliable space-time congruency effect in Russian native speakers (Blinded for review). The stimuli were matched for their letter length across both the past/future subset (*t*(22) = –1.14, *p* = .267; past-related words: *M* = 7.72, *SD* = 2.02; future-related words: *M* = 8.42, *SD* = 2.27) and the time-related/neutral subset (*t*(46) = 0.05, *p* = .935; time-related words: *M* = 7.92, *SD* = 2.17; neutral words: *M* = 7.92, *SD* = 2.26). Also, the words were matched for their lemma frequency^1^ (log10) across both the past/future subset (*t*(22) = –0.44, *p* = .663; past-related words: *M* = 1.47, *SD* = 0.71; future-related words: *M* = 1.58, *SD* = 0.58) and the time-related/neutral subset (*t*(46) = –0.07, *p* = .946; time-related words: *M* = 1.53, *SD* = 0.63; neutral words: *M* = 1.51, *SD* = 0.50). Both stimulus types included nouns, adverbs, adjectives, and participles in similar proportions. Nevertheless, we controlled for stimulus type in the statistical analyses (see Data preprocessing and statistical analyses for details).

**Table 1:** Translation of words included in the stimulus material.


	TIME-RELATED WORDS		NEUTRAL WORDS	
	
	ORIGINAL	TRANSLATION	ORIGINAL	TRANSLATION

**Past**	былое	*bygone days*	анализ	*analysis*

	вчера	*yesterday (adv)*	аппарат	*apparatus*

	вчерашний	*yesterday’s (adj)*	бензин	*petrol*

	давно	*long ago*	биология	*biology*

	издавна	*since long ago*	вовсе	*at all*

	издревле	*since ancient times*	вообще	*generally*

	минувший	*bygone*	воспитание	*upbringing*

	недавно	*recently*	вроде	*seemingly*

	позавчера	*the day before yesterday*	обычный	*usual*

	прошлогодний	*last year’s*	существо	*being*

	прошлое	*the past (n)*	глобализация	*globalisation*

	прошлый	*past (adj)*	экономика	*economics*

**Future**	будущее	*the future (n)*	деревянный	*wooden*

	будущий	*upcoming*	стеклянный	*glass*

	вскоре	*Shortly*	стиль	*style*

	грядущее	*what is to come*	продукт	*product*

	грядущий	*future (adj)*	также	*also*

	завтра	*tomorrow (adv)*	теоретический	*theoretical*

	завтрашний	*tomorrow’s (adj)*	учитель	*teacher*

	наступающий	*forthcoming*	факультет	*faculty*

	планируемый	*planned*	чемпионат	*championship*

	послезавтра	*the day after tomorrow*	характер	*temperament*

	предстоящий	*impending*	цивилизация	*civilisation*

	скоро	*soon*	фильтр	*filter*


All words were randomly presented in capital letters in the center of the screen (text height: 80 px). Participants were instructed to categorize the stimuli as either time-related or neutral by pressing the right and left lateral response keys. The instructions were systematically varied across blocks: in one block, time-related words were classified using the left key and neutral words – using the right key, and in the other block, this stimulus-response combination was reversed. The order of the two blocks was counterbalanced across participants. As a result, the combinations of time-related words and response keys were rendered as either congruent for the time-related words (left – past, right – future) or incongruent (left – future, right – past). For neutral words, left vs. right key distinction was not relevant, and these words were used as a control condition.

### 2.3. Procedure

**Figure 2:**
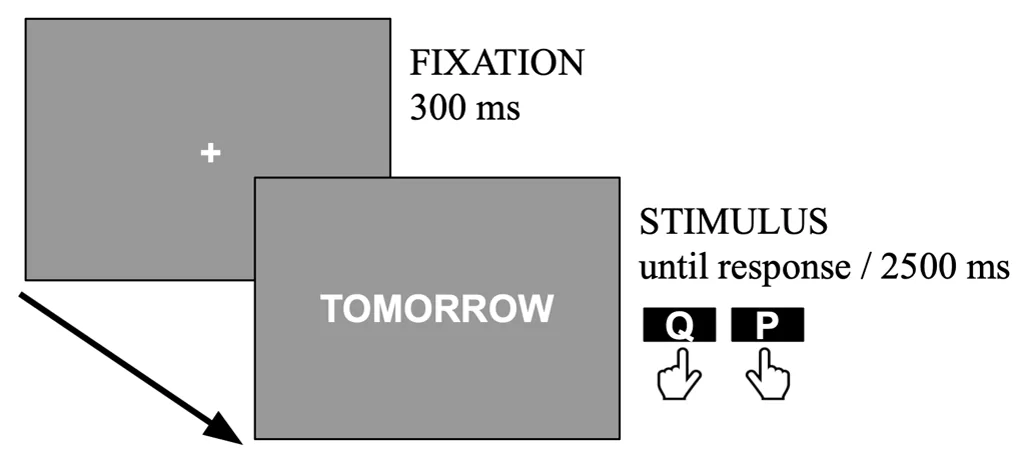
Example of an experimental trial sequence. The stimulus example is translated to English for clarity. Not to scale.

The experiment was conducted online using Gorilla Experiment Builder (www.gorilla.sc; Anwyl-Irvine et al., 2021). In order to maximize data quality, participants were asked to sit in a quiet room, avoid distractions during the experiment, close all other software, and switch to full-screen mode. After completing the demographics questionnaire, the main experiment started, which involved two blocks, each with randomized stimulus presentation. A short break between blocks was included to reduce fatigue. Each block started with four practice trials using stimuli not included in the main experiment, with feedback provided. Each trial began with a centrally presented fixation cross that stayed on the screen for 300 ms. Following the fixation cross, a word appeared in the center of the screen. Participants were instructed to read the word and categorize it as either time-related or not by pressing the left or right keyboard keys using left and right index fingers (*Q* and *P* keys on US keyboard layout, respectively) in accordance with the block’s instruction. The word remained on the screen until a response was given but no longer than 2500 ms, immediately after which a new trial began with no additional inter-trial interval. If participants incorrectly classified the word or did not respond within the 2500 ms timeout period, the trial was coded as incorrect. Each word was presented randomly once in each of the two blocks, resulting in a total of 96 unique trials. A typical trial sequence is shown in Figure 2. No feedback was given during the main part of the experiment.

### 2.4. Data preprocessing and statistical analyses

Statistical analyses were conducted using the R software package version 4.1.3 (RStudio Team, 2022). Since the average accuracy performance across participants was near ceiling (95.8%), the accuracy was not analyzed further. The main dependent variable was Reaction Time (RT), which was defined as the time from the onset of the word to the key press, measured in milliseconds. We used a design with the following independently manipulated experimental factors: Word type (past/neutral/future), Response key (left/right), Age (continuous within 19 to 80 range), and Physical activity (continuous on a scale 1 to 6). For data trimming, incorrect responses and trials without responses, including delays over the timeout period, were excluded (4.2% of the data). For data normalization, a logarithmic transformation (log10(RT)) was applied. A Linear Mixed Effects Model (LMM) analysis was then conducted on this measure using the *lme4* package (Bates et al., 2015). The initial model comprised Word type (past/neutral/future), Response key (left/right), Age (continuous), and Physical activity (continuous), as well as interactions between these factors. The categorical predictor Response key was assigned sum-coded contrasts (– 0.5 and 0.5) (Barr et al., 2013). The Word type predictor was coded as –1 for past-related words, 0 for neutral words, and +1 for future-related words. Continuous predictors Age and Physical activity were mean-centered. Participants and stimuli were indicated as random effects. The initial model was: “log10(RT) ~ 1 + Word type * Response key * Age * Physical activity + (1 + Word type * Response key | Participant) + (1 + Word type * Response key | Stimulus)”. Backwards elimination using the *drop1* function from the *lme4* package was employed to identify the best-fitting model. Effects and interactions that did not improve model fit (*p* ≥ .100) were successively eliminated.

## 3. Results

All factors (Word type, Response key, Age, and Physical Activity), as well as a four-way interaction between them, remained in the model after backwards elimination. The final model was: “log10(RT) ~ 1 + Word type * Response key * Age * Physical activity + (1 + Word type + Response key | Participant) + (1 + Response key | Stimulus)”. Marginal *r*-squared (variance explained by fixed effects only) was .120, and conditional *r*-squared (variance explained by the whole model) was .485. Results are presented in Table 2. Below, we discuss only significant (*p* <.05) effects and interactions of primary interest. All the figures represent raw RT data for a better visualization.

**Table 2:** Statistical results: Output of the best-fitting linear mixed-effects model.


RANDOM EFFECTS	NAME	VARIANCE	SD		

Participants	Intercept	0.0039	0.0622		

	Word type	0.00001	0.0085		

	Response key	0.0002	0.0147		

Stimuli	Intercept	0.0020	0.0445		

	Response key	0.0006	0.0252		

Residual		0.0086	0.0928		

**FIXED EFFECTS**	***B***	***SE***	***CI***	***t*-value**	***p*-value**

Intercept	2.8874	0.0093	2.8692 – 2.9056	310.95	<.001

Word type	0.0022	0.0092	–0.0160 – 0.0203	0.23	.815

Response key	0.0012	0.0045	–0.0076 – 0.0099	0.26	.795

**Age**	**0.0022**	**0.0004**	**0.0015 – 0.0030**	**6.11**	**<.001**

Physical activity	0.0065	0.0049	–0.0030 – 0.0160	1.34	.181

Word type * Response key	–0.0043	0.0059	–0.0160 – 0.0073	–0.73	.467

Word type * Age	**0.0002**	**0.0001**	**0.0000 – 0.0004**	**2.32**	.020

Response key * Age	0.0002	0.0001	–0.0001 – 0.0005	1.36	.175

Word type * Physical activity	–0.0003	0.0013	–0.0027 – 0.0022	–0.22	.829

Response key * Physical activity	–0.0031	0.0019	–0.0068 – 0.0006	–1.62	.104

Age * Physical activity	–0.0004	0.0003	–0.0010 – 0.0002	–1.20	.229

Word type * Response key * Age	0.0000	0.0002	–0.0003 – 0.0004	0.28	.777

Word type * Response key * Physical activity	–0.0003	0.0022	–0.0046 – 0.0039	–0.15	.884

Word type * Age * Physical activity	**–0.0002**	**0.0001**	**–0.0003 – –0.0000**	**–2.23**	.026

Response key * Age * Physical activity	–0.0001	0.0001	–0.0003 – 0.0002	–0.44	.658

Word type * Response key * Age * Physical activity	**–0.0004**	**0.0001**	**–0.0006 – –0.0001**	**–2.72**	.006


**Figure 3:**
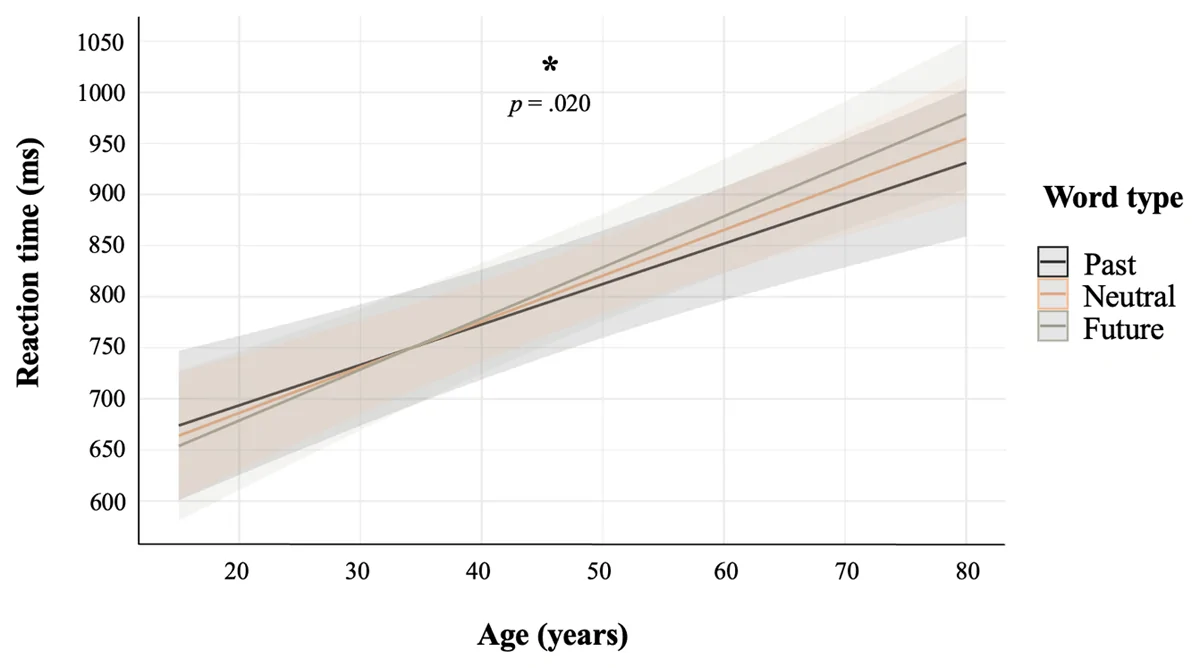
Interaction between Word type and Age (model-estimated RT data). Observed per-participant mean RTs are presented in the Supplementary Materials (Figure S1).

First, our analysis registered a statistically significant main effect of Age (*b* = 0.0022, *t* = 6.11, *p* < .001): the higher the participants’ age was, the slower they performed the task. More importantly, our analysis revealed a reliable interaction between Word type and Age (*b* = 0.0002, *t* = 2.32, *p* = .020): Younger participants processed future-related words faster than past-related words, whereas senior participants processed past-related words faster than future-related words (see Figure 3).

**Figure 4:**
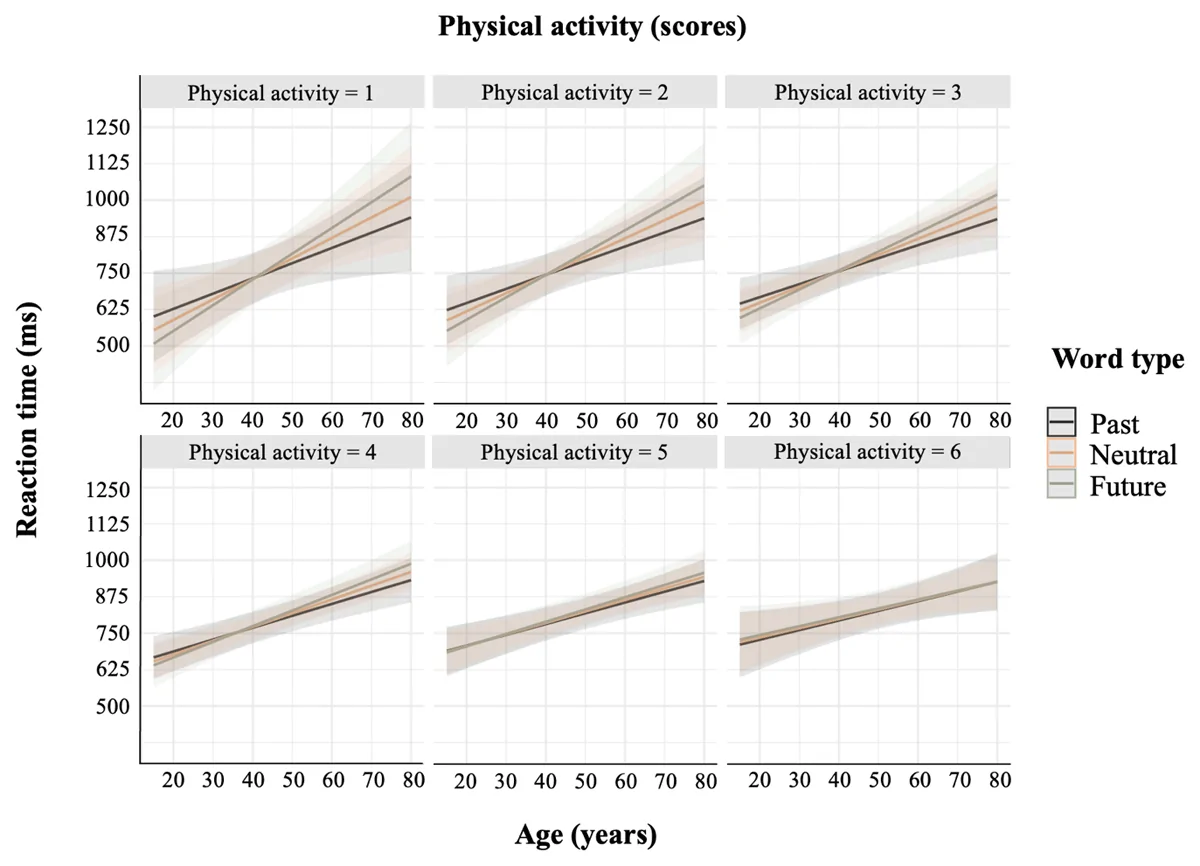
Interaction between Word type and Age along Physical activity (scores) (model-estimated RT data). *Note*. The model-based predictions are shown separately for each observed level of Physical activity scores: 1 (N = 5), 2 (N = 8), 3 (N = 10), 4 (N = 15), 5 (N = 41), and 6 (N = 15).

Moreover, the model revealed a reliable three-way interaction between Word type, Age, and Physical activity (*b* = –0.0002, *t* = –2.23, *p* = .026). The main analysis constitutes our primary and most informative test of this interaction, as it preserves the full information available in Age and Physical Activity variables and avoids the statistical power loss inherent in any form of dichotomization (Altman & Royston, 2006; Maxwell & Delaney, 1993). To further explore and illustrate the nature of this three-way interaction, we conducted two additional analyses. First, we examined the two-way interaction between Word type and Age across different levels of Physical activity. Visualization of the model revealed that as self-reported level of physical activity increased, the age-related differences in processing past, future, and neutral words diminished (Figure 4). To validate this pattern statistically, we also performed a follow-up analysis focusing on participants at the extremes of the Physical Activity distribution. We categorized participants into low (levels 1–3; N = 23) and high (levels 5–6; N = 56) Physical activity groups, excluding the middle group (level 4, N = 15) from this particular analysis. While dividing a continuous variable into categories can result in information loss (Royston et al., 2006), we implemented this approach as a complementary step to our main analysis rather than as the primary basis for interpretation.^2^ We specifically targeted the extremes of the distribution because this method allows for testing conditions where the modulation effect is most versus least pronounced, whereas a simple mean/median split might obscure the gradient by combining participants closer to the average value (e.g., McClelland et al., 2015). For this analysis, we created a new binary predictor for Physical activity (low/high), which was assigned sum-coded contrasts (–0.5 and 0.5). These data were then submitted to a linear mixed model analysis with the same factors and interactions as in the main analysis. Confirming the pattern from the main analysis, our follow-up analysis revealed that the differences between past, future, and neutral word processing were modulated by participant’s age (the interaction between Word type and Age) only in the lower physical activity group (*b* = 0.0005, *SE* = 0.0002, *t* = 3.14, *p* = .002), but not in the higher physical activity group (*b* = 0.0001, *SE* = 0.0001, *t* = 0.52, *p* = .603). Specifically, in the lower physical activity group, younger participants processed future-related words faster than past-related words, whereas older participants processed past-related words faster than future-related words.

**Figure 5:**
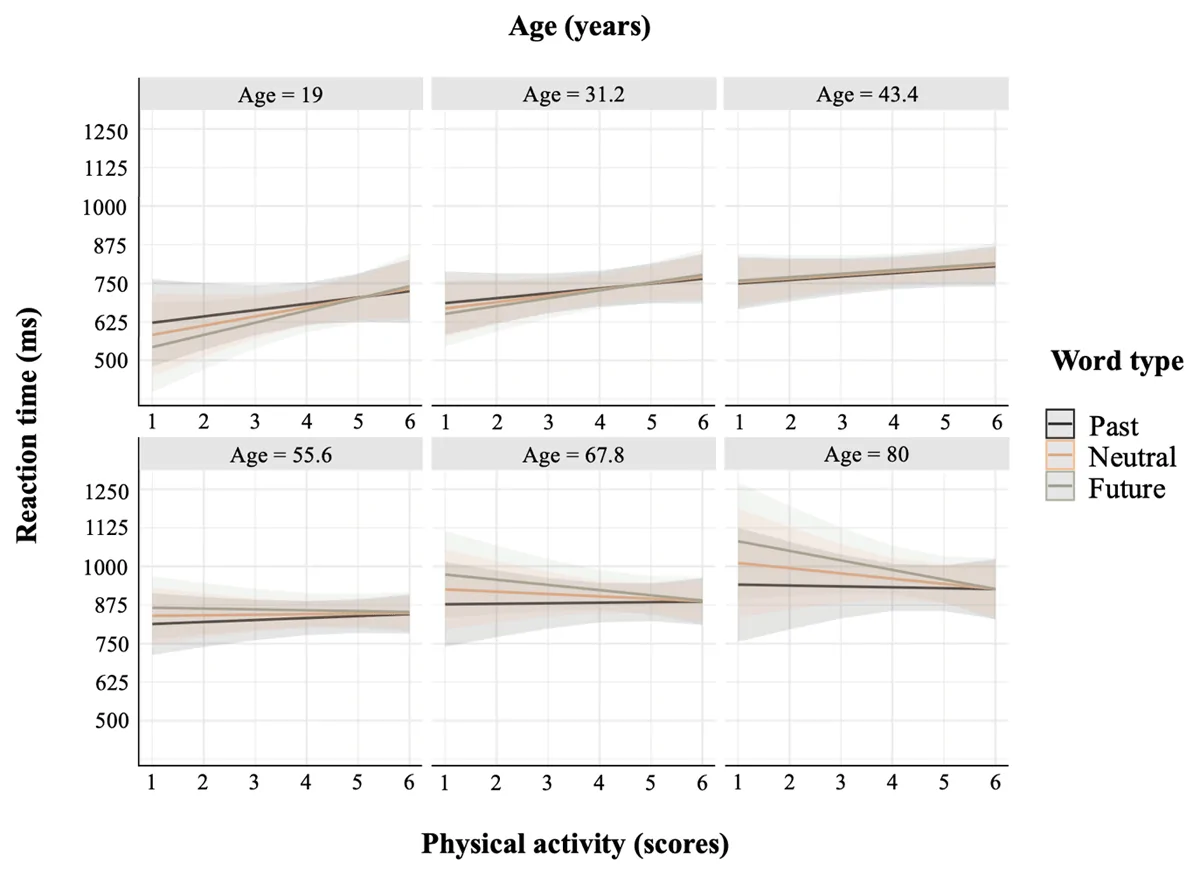
Interaction between Word type and Physical activity along Age (years) (model-estimated RT data). *Note*. The model-based predictions are evaluated at six illustrative, evenly spaced ages across the entire sample range (19–80). There is no partitioning of participants into discrete age groups, since predictions at each age value are informed by data from the entire sample (with stronger influence from participants whose ages are close to the probed value).

We then examined the three-way interaction by breaking it down into a two-way interaction between Word type and Physical activity along the Age variable. Model visualization indicated that self-reported level of physical activity had a more pronounced effect on reducing the differences between word processing (the interaction between Word type and Physical activity) in older adults compared to younger ones (Figure 5). To statistically test this pattern, we conducted a follow-up analysis at the extreme ends of the Age distribution. We first divided the full sample into tertiles based on participants’ ages, yielding cutoffs at 36 years (lower tertile boundary) and 60 years (upper tertile boundary). After this, we excluded the middle tertile and retained the lower and upper extremes. This resulted in a nearly equal number of younger (N = 31, age from 19 to 34; Mean = 29.1) and more senior participants (N = 32; age from 60 to 80; Mean = 68.4). This new categorical predictor was coded as a binary factor (young vs. senior) and assigned sum-coded contrasts (–0.5 and 0.5). These data were submitted to a linear mixed model analysis with the same factors and interactions between them as in the main analysis. As in the case with the previous follow-up analysis, this analysis is intended solely as a complementary illustration of the direction of the effect established in the primary continuous analysis, and should not be interpreted as *independent* evidence. Complementing the pattern from the main analysis, the new analysis revealed that the difference between the processing of past, future, and neutral words was modulated by physical activity (the interaction between Word type and Physical activity) only for older participants (*b* = –0.0081, *SE* = 0.0028, *t* = –2.93, *p* = .003), but not for younger participants (*b* = 0.0016, *SE* = 0.0020, *t* = 0.80, *p* = .426): The higher the self-reported level of seniors’ physical activity was, the smaller the differences between processing word categories became.

**Figure 6:**
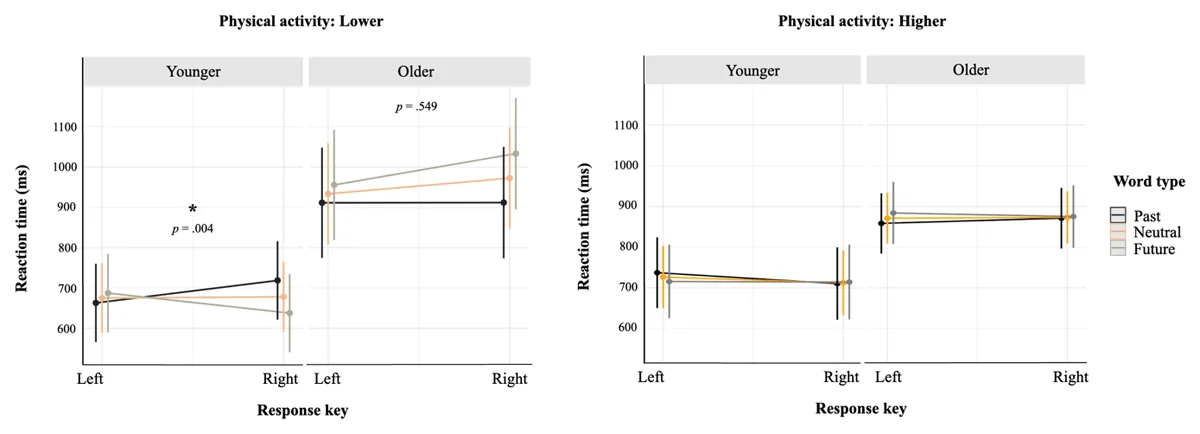
Interaction between Word Type and Response Key: Releveling by Physical activity and Age (model-estimated RT data). Error bars represent standard errors.

Finally, our analysis revealed a reliable four-way interaction between Word type, Response key, Age, and Physical activity (*b* = –0.0004, *t* = –2.72, *p* = .006). To further examine this interaction, we first re-levelled the dataset separately for each of the two physical activity groups obtained at the previous step. Modulation of the space-time congruency effect (i.e., the interaction between Word type and Response key) by Age was found only in the lower physical activity group (*b* = 0.0008, *SE* = 0.0003, *t* = 2.62, *p* = .009), but not in the higher physical activity group (*b* = –0.0002, *SE* = 0.0002, *t* = –1.14, *p* = .255). In order to further scrutinize the results specifically for the lower physical activity group, we split the data into the two age groups we had calculated in the previous analysis. This analysis registered a reliable space-time congruency effect in younger participants (*b* = –0.0254, *SE* = 0.0087, *t* = –2.92, *p* = .004) but not in older ones (*b* = 0.0080, *SE* = 0.0133, *t* = 0.60, *p* = .549) (see Figure 6): For younger participants, future-related words were processed faster with the right response key, whereas past-related words were processed faster with the left response key.

## 4. Discussion

The present study examined how individuals’ bodily and life-course experiences affect their processing of temporal concepts. Specifically, we investigated whether participants’ age and self-reported level of physical activity influence their spatial–temporal mapping and the speed with which they process past- and future-related words. To this end, we asked participants ranging from 19 to 80 years old to read past- and future-related words as well as control neutral stimuli and classify them as time-related or unrelated by pressing left and right response keys in a counterbalanced fashion. Space–time congruency effects were registered as RT differences between congruent (past + left, future + right) and incongruent (past + right, future + left) conditions, with neutral word responses serving as a control. To the best of our knowledge, this is the first study to directly compare horizontal space–time congruency effects in younger and older participants whilst simultaneously taking into account individuals’ self-reported physical activity level.

We obtained three main findings. First, older participants were overall slower in the semantic categorisation task than their younger counterparts. Second, participants’ age modulated their relative speed of past versus future word processing, with younger participants responding faster to future-related words and older participants responding faster to past-related words. Third, this age-related asymmetry was itself modulated by physical activity, and space–time congruency effects showed an age-related decline specifically in the lower physical activity group. Below, we will briefly discuss each of these findings before considering their broader theoretical implications, limitations, and directions for future research.

### 4.1. General slowing

Our first finding – that older participants showed overall slower performance – is consistent with a large body of research documenting age-related increases in response times across a wide range of cognitive tasks (Park et al., 2002; Park & Festini, 2017). This general slowing likely reflects broad neurocognitive changes accompanying ageing, including reductions in processing speed, attentional capacity, cognitive control (Brito et al., 2023; Lustig et al., 2007; Murman, 2015) as well as motor abilities. In the domain of semantic processing specifically, older adults show greater difficulty relative to younger adults in a range of tasks involving lexical access and word categorisation (Hoffman, 2018; Meyer & Federmeier, 2010; Wu & Hoffman, 2022; Zhu et al., 2019), and our results extend this pattern to the temporal semantic domain. Importantly, however, overall slowing does not in itself explain the differential pattern of past versus future word processing observed in our second finding, which requires a more specific account in terms of age-related changes in temporal cognition.

### 4.2. Age-related shift in temporal word processing

Our second and theoretically central finding is that age modulated the relative speed of processing past- versus future-related words: younger participants processed future-related words faster than past-related words, whereas older participants showed the reverse pattern, responding more quickly to past-related words. This finding is consistent with, and extends, the results of Anelli and colleagues (2016). They reported that older adults were slower than younger adults, specifically when classifying events under a future self-location condition, suggesting a reduced capacity for future self-projection. Our study goes beyond this earlier work by demonstrating, for the first time, a clear RT difference between past and future word processing as a function of age in a standard semantic categorisation paradigm – a contribution that is highly informative because reaction times provide an objective, millisecond-precise index of processing fluency that is not subject to the introspective biases inherent in verbal or rating-based measures.

The most parsimonious explanation for this pattern draws on the well-established age-related shift in temporal orientation. As people age, the perception of a shrinking future and an expanding experienced past leads to a reorientation of attention and cognitive resources toward past-related information (Kooij et al., 2018; Strough et al., 2016). Older adults show higher levels of retrospection and lower levels of future-oriented thinking compared to younger adults (Irish et al., 2019), are more responsive to past-oriented than future-oriented advertising (Imtiaz & Ji, 2021), and generate richer episodic detail for past than future events (Addis et al., 2008, 2010). According to socioemotional selectivity theory and related frameworks, this shift is partly motivated by a perceived contraction of future time: as the future horizon shortens, investment in future-oriented goals and processing decreases relative to consolidation and reflection on the past (Carstensen, 2006; Heckhausen & Krueger, 1993). In our study, this reorientation is reflected behaviourally in a facilitation effect for past-related words in older participants – faster responses to past than future words – consistent with the idea that attentional and semantic resources are more readily engaged by temporally proximal, past-focused content in this age group. This interpretation is further supported by evidence that individual differences in time perspective influence the processing of time-related language more broadly, including the use and interpretation of words referring to short- versus long-term intervals (Waliński, 2014), and that future time perspective is associated with enhanced episodic future thinking, which may in turn facilitate processing of future-related lexicon (Arnold & Szpunar, 2015).

### 4.3. Modulation by physical activity

Our third finding is that the age-related asymmetry in past versus future word processing was significantly modulated by participants’ self-reported level of physical activity. The past–future RT difference was present only among participants in the lower physical activity group, where younger participants processed future-related words faster, whereas older participants were faster in processing past-related words. Strikingly, this age-related divergence was absent in the higher physical activity group, where past and future word processing did not differ significantly as a function of age. Moreover, within older participants, higher self-reported levels physical activity was associated with smaller differences in the processing of past and future words, suggesting a dose-dependent buffering effect.

These results are consistent with a growing body of evidence showing that physical activity supports cognitive functioning in older adults, including episodic memory, executive control, and processing speed (Aghjayan et al., 2022; Hamm et al., 2024; Sewell et al., 2023). Crucially, physical activity has also been linked specifically to the maintenance of future time perspective in older age: higher activity levels are associated with a more extended future horizon (Kooij et al., 2018; Rutt & Löckenhoff, 2016), greater investment in long-term health goals (Kooij & Van De Voorde, 2011), and a more optimistic orientation toward the future (Daskalopoulou et al., 2017). It is therefore plausible that physically active older adults retain a more balanced temporal orientation – one less dominated by past-focused attention – and that this is reflected in their more “youth-like” pattern of temporal word processing. In this account, physical activity does not directly influence the lexico-semantic representations of past and future words per se, but rather modulates the broader attentional and motivational context within which temporal concepts are processed, preserving the future-oriented bias that characterises younger adults’ performance.

It is important, however, to interpret these findings with appropriate caution. Physical activity in the present study was assessed via a single self-report item asking how often participants engage in physical activity, without specifying the type, intensity, or duration of exercise. This measure is necessarily coarse and may be subject to individual differences in how “physical activity” is interpreted – for instance, some participants may have had gym-based exercise in mind, while others may have included everyday walking or occupational physical demands. As such, the present results are best regarded as preliminary and exploratory with respect to physical activity, and the variable is most appropriately treated as a broad proxy for general physical well-being rather than as a direct measure of exercise behaviour. Future studies should employ validated, multi-item measures of physical activity or objective measures (e.g., accelerometry) to examine these effects with greater precision. Notwithstanding these limitations, the convergence of our findings with the broader literature on physical activity and cognitive ageing lends them a degree of plausibility that warrants further investigation.

### 4.4. Space–time congruency effects and their age-related decline

A different facet of our findings concerns the space–time congruency effect itself. As described above, we found that congruency effects – faster responses when past maps to the left and future maps to the right – were present in younger participants with lower self-reported levels of physical activity but absent in their older counterparts in the same group. This age-related effect was not observed in the higher physical activity group. The results partially extend those of Anelli and colleagues (2016) while also qualifying them: Whereas Anelli et al. found age-related slowing specifically in a future self-location condition, our results suggest a broader age-related attenuation of spatial–temporal mapping at the level of response selection, at least under conditions of lower physical activity.

One interpretation of this pattern, consistent with a mental time travel framework, is that age-related decline in hippocampal function and episodic memory reduces the vividness and accessibility of the past and future mental scenarios that support spatial–temporal mappings (Coste et al., 2012; Greene & Naveh-Benjamin, 2023; Lister & Barnes, 2009). On this view, the horizontal MTL depends, at least partly, on the ability to mentally project oneself into the past or future, and this projective capacity is diminished in older adults with lower physical activity whose cognitive resources may be less well preserved. Physical activity, in turn, may help maintain the episodic memory and executive functioning that underlie this capacity, thereby preserving space–time congruency effects in more active older adults (Aghjayan et al., 2022; Hamm et al., 2024).

However, an important alternative explanation must also be considered. Recent work by Scozia and colleagues (2023a, 2023b, 2024) has demonstrated that STEARC effects do not reflect the automatic activation of a spatial mental representation of time but rather emerge as a late heuristic at the level of response selection, appearing preferentially at slower response latencies and depending on the joint recruitment of spatial and temporal codes by task demands. Under this account, the reduced congruency effects observed in older low-activity participants might not reflect weakened spatial representations of time per se. Instead, since older adults respond more slowly overall, the distribution of their RTs falls in a different region of the latency spectrum, potentially altering the conditions under which spatial coding of time is recruited. In other words, age differences in congruency magnitude may partly be a consequence of differences in the shape and location of RT distributions rather than of any qualitative change in how time is spatially represented. This alternative deserves serious consideration and points to the importance of analyzing congruency effects as a function of RT in future ageing studies, rather than simply comparing mean congruency scores across age groups.

### 4.5. Theoretical implications: dynamic conceptual time

Taken together, our findings contribute to an emerging picture of temporal cognition as fundamentally dynamic – shaped not only by cultural and linguistic factors, but by ongoing developmental and experiential processes across the lifespan. We propose the construct of dynamic conceptual time to capture the idea that individuals’ spatial–temporal mappings and temporal processing biases are not fixed properties but rather evolve continuously under the influence of ageing, physical activity, and related experiential factors. Purportedly, one’s MTL is just taking shape during development (Nava et al., 2018), being influenced by a variety of factors as a child learns to speak and write (Bergen & Lau, 2012; Ouellet et al., 2010b; Pitt & Casasanto, 2020), to use calendars (Starr & Srinivasan, 2021) and situational primes (Tillman et al., 2018).

The dominant mapping type begins to form in childhood after the onset of formal schooling (Starr & Srinivasan, 2021; Tillman et al., 2018), but it does not remain stable in adulthood, with potential changes in MTL direction taking place – for instance, the future becomes perceived as either ahead or behind the observer, depending on religious beliefs (Li & Cao, 2018b), political attitudes (Li & Cao, 2022), stressful events (Li, 2021), pregnancy (Li & Cao, 2018a), and time of the day (Li, 2018). A person can also learn a second language and acquire a new type of spatial-temporal mapping typical of that language (Miles et al., 2011). Moreover, different contexts, lifestyles, and personality traits can influence the perception of time as moving toward the ego or as the ego moving through time (e.g., “I am approaching the deadline” versus “the deadline is approaching”; Lai & Boroditsky, 2013; see Feist & Duffy, 2023 for review). Interestingly, individuals who adopt ego-moving time perspective show a higher level of extroversion, procrastination, and resilience (Duffy & Feist, 2014; Qin, 2023). Finally, with ageing, the mapping of time changes too: The past begins to be perceived as being behind and the future as ahead of the observer (Bylund et al., 2020; de la Fuente et al., 2014; Núñez & Sweetser, 2006). The present study adds to this list of modulating factors, demonstrating the influence of ageing and self-reported level of physical activity on the processing of temporal concepts. Crucially, this influence penetrates deep into the neurocognitive system, affecting not merely the time processing or spatial mapping but even the linguistic codes, i.e., lexico-semantic representations of time-related information in our brain.

From a broader perspective, age-related differences in temporal word processing may carry practical significance beyond their theoretical interest. Temporal orientation – the balance between past- and future-directed cognition – is a consistent predictor of well-being, health behaviour, and adaptive functioning (Gabrian et al., 2017). Future-oriented thinking is associated with greater life satisfaction (Corlett & MacLeod, 2021), prosocial behaviour (Baumsteiger, 2017), responsible financial decision making (Joireman et al., 2005), and effective health self-management (Schacter et al., 2017). Conversely, a shift toward past-focused cognition, of the kind observed in our older low-activity participants, may represent an early behavioural marker of broader cognitive and motivational changes associated with less healthy ageing. Notably, the differences in our data began to emerge at as early as 35–40 years of age, even in a sample that was relatively active and engaged with health and educational programmes. This suggests that the population-level impact of these changes may be considerably larger, particularly among individuals who are less engaged in health-promoting activities or who live in resource-limited contexts. *Dynamic conceptual time* – the evolving pattern of temporal processing across the lifespan – may, therefore, be a useful construct for future research on subjective time perception, alongside established parameters such as time attitude, temporal self-location, and perceived speed of time (Gabrian et al., 2017; Rutt & Löckenhoff, 2016).

### 4.6. Limitations and future directions

Several limitations of the present study warrant discussion. First, with 94 participants (over double that of previous similar studies; see Methods), the study provides 78.5% power to detect the four-way interaction between Word type, Response key, Age, and Physical activity factors – whilst high, it is still marginally below the conventional 80% target. Post-hoc power simulations suggest that a sample of approximately 105 participants would achieve adequate power, and we recommend that future studies target this or a larger sample size, particularly given the complexity of the design. Second, as discussed above, physical activity was measured by a single self-report item, which limits the precision and validity of this variable. Future studies should use validated multi-item questionnaires and/or objective measures. Third, the task required participants to classify words as time-related or neutral rather than as explicitly past- or future-related, which may have attenuated space–time congruency effects relative to tasks requiring direct past/future judgements (Shaki & Fischer, 2023; von Sobbe et al., 2019; Kühne et al., 2025). Replication using an explicit temporal categorisation task (Santiago et al., 2007; Torralbo et al., 2006) would help clarify whether the present pattern of results generalises across task formats. Fourth, to enable a clearer stimulus contrast, present-related words were not included in the stimulus set, precluding examination of present-focused shifts that have been reported in some older adult samples (Mello et al., 2022). A fuller stimulus set spanning past, present, and future would allow more fine-grained analysis of age-related changes in temporal attention. Finally, future research would benefit from including a broader range of lifestyle and well-being measures – such as social activity, marital status, sleep quality, and life satisfaction – to better characterise various experiential factors that may modulate dynamic conceptual time across the lifespan.

## 5. Conclusion

To conclude, the present study reveals age-related differences in the speed of processing past- and future-related words: Younger participants processed future-related words faster than past-related words, whereas older participants processed past-related words faster than future-related words. Notably, a higher self-reported level of physical activity was an important factor in reducing differences in processing these word categories in senior participants. Moreover, we demonstrated an age-related decline in space-time congruency effects for participants with lower self-reported levels of physical activity. To our knowledge, the present study is the first to demonstrate ageing effects both in the spatial–temporal mapping at the level of response selection and in past and future word processing per se. These results indicate that the subjective perception of time undergoes life-long changes influenced by multiple proxy factors, including ageing and physical activity. Our findings suggest an integrative construct of *dynamic conceptual time* whereby the individual differences in physical and developmental experience lead to gradual changes in their processing of temporal concepts.

## Supplementary Materials

**Figure S1:**
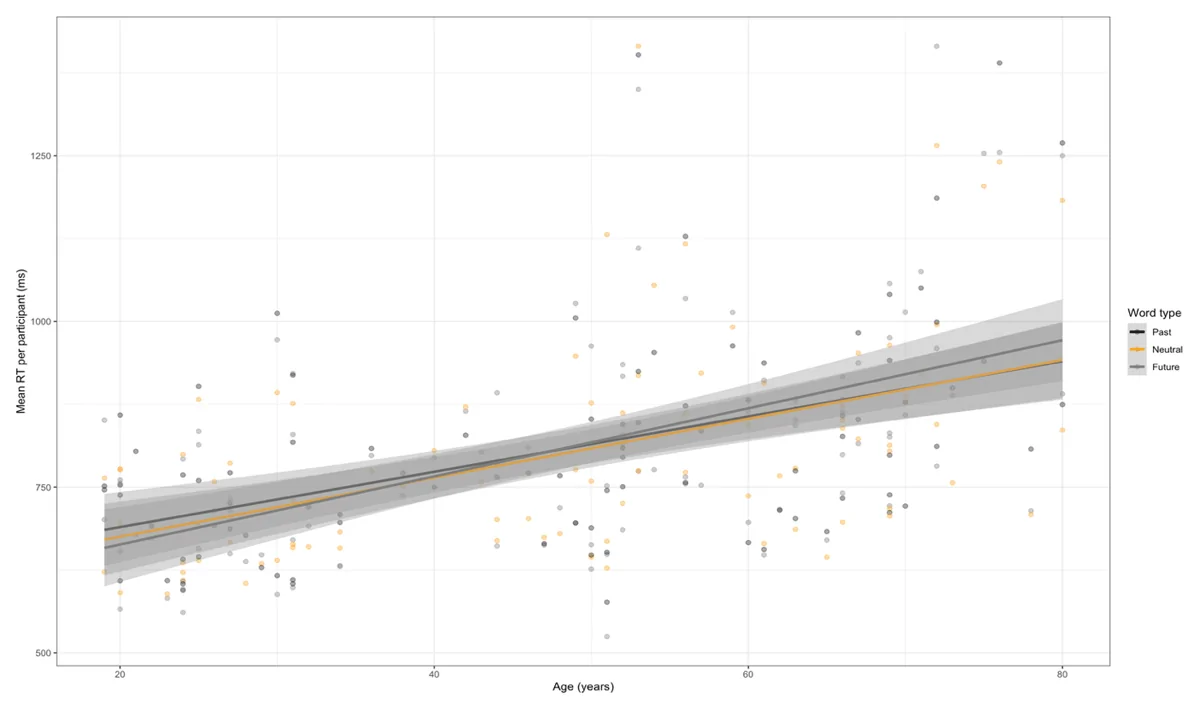
Observed mean reaction times (ms) as a function of Age and Word type. *Note.* Each data point represents a single participant’s mean RT for a given word type (past, neutral, future), plotted at their actual age.

## Data Availability

The datasets generated for this study and processing scripts can be found in the Open Science Framework (OSF) at this link.

## Footnotes

Lemma frequency is the cumulative frequency of all inflected word forms that belong to a single dictionary headword (lemma).

We do recognize that such grouping is limited in nature: the asymmetry in group composition reflects the natural distribution of responses in our sample rather than a theoretically motivated boundary, and different cutpoints could in principle yield different results. We therefore caution against over-interpreting this binary analysis independently of the main – continuous – model, which remains the primary basis for our conclusions.
